# Characterization of isolated late preantral and early antral equine ovarian follicles

**DOI:** 10.1530/RAF-25-0109

**Published:** 2026-03-25

**Authors:** Ana Normélia P Morais, Gustavo D A Gastal, Francisco L N Aguiar, Kendall A Hyde, Melba O Gastal, Laritza F Lima, Lucy V S Ñaupas, Ana Flávia B Silva, Verónica A B Becerra, Luiza Gheno, Bruna R Curcio, Carine D Corcini, Ana Paula R Rodrigues, José R Figueiredo, Antonio S Varela, Eduardo L Gastal

**Affiliations:** ^1^ Animal Science, School of Agricultural Sciences, Southern Illinois University, Carbondale, Illinois, USA; ^2^ Laboratory of Manipulation of Oocytes and Preantral Follicles, Faculty of Veterinary Medicine, State University of Ceará, Fortaleza, Ceará, Brazil; ^3^ Instituto Nacional de Investigación Agropecuaria, Estación Experimental INIA La Estanzuela, Colonia, Uruguay; ^4^ Department of Veterinary Medicine, Sousa Campus, Federal Institute of Education, Science and Technology of Paraíba, Sousa, Paraíba, Brazil; ^5^ Facultad de Ciencias Agropecuarias, Carrera de Medicina Veterinaria, Universidad Técnica de Ambato, Ambato, Tungurahua, Ecuador; ^6^ Department of Veterinary Clinics, College of Veterinary Medicine, Federal University of Pelotas, Pelotas, Rio Grande do Sul, Brazil; ^7^ Department of Comparative Animal Reproduction, Institute of Biological Sciences, Federal University of Rio Grande, Rio Grande, Rio Grande do Sul, Brazil

**Keywords:** mare ovary, microdissection, follicular viability, spatial distribution, histone trimethylation, folliculogenesis

## Abstract

**Graphical Abstract:**

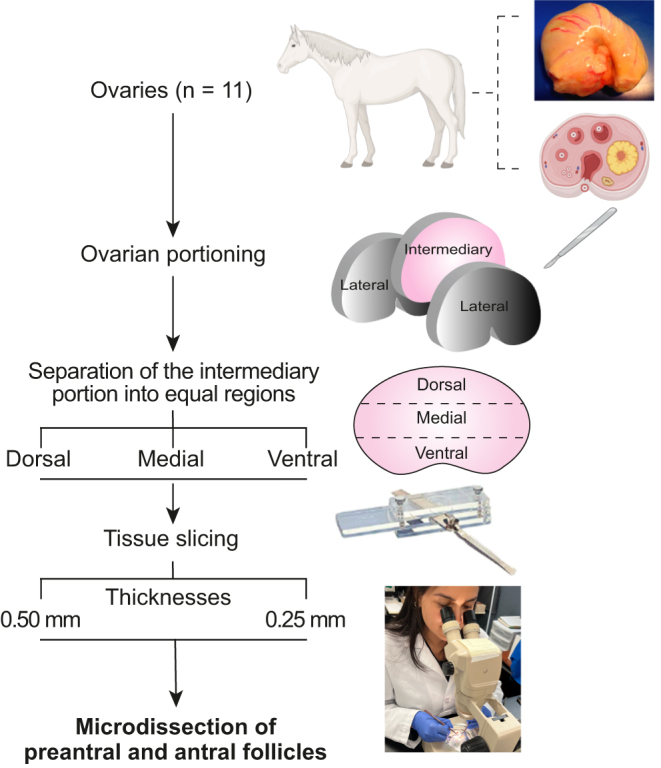

**Abstract:**

Understanding how to effectively isolate a large number of healthy follicles is essential for increasing success rates in ARTs, such as *in vitro* culture and cryopreservation. Late secondary (preantral) and early tertiary (antral) follicles isolated from the intermediary portion of equine ovarian tissue sliced at two different thicknesses (0.25 vs 0.50 mm) were characterized according to regional distribution (dorsal, medial, and ventral), morphofunctional features, and epigenetic profiles. After isolation via microdissection, follicles were evaluated for membrane integrity (trypan blue), viability (propidium iodide and Hoechst 33342), mitochondrial membrane potential (JC-1), reactive oxygen species (ROS; H2DCFDA), and histone trimethylation patterns (H3K4me3 and H3K9me3). Regardless of the ovarian tissue slice thickness or region, all isolated follicles had intact membranes, confirming the effectiveness of mechanical microdissection. The key findings from this study reveal that i) recovery of preantral and antral follicles within different ovarian regions is influenced by tissue slicing thickness; ii) the abundance of recoverable follicles varies among the ovarian regions; iii) preantral and antral follicle viability and ROS levels remain consistent between developmental stages, irrespective of tissue slicing thickness, although mitochondrial membrane potential is influenced by slicing thickness; iv) in antral follicles, viability and ROS levels vary depending on location within the ovarian regions; and v) epigenetic markers (H3K4me3 and H3K9me3) exhibit different patterns, depending upon follicular developmental stage. Altogether, these results demonstrate that optimal follicle isolation depends on tissue thickness and ovarian region and that viable, good-quality preantral and antral follicles can be successfully isolated from equine ovarian tissue.

**Lay summary:**

Follicles are the functional units of the ovary that contain and support the egg. Depending on the presence of a fluid-filled cavity called an antrum, follicles are developmentally classified as preantral (no antrum) or antral (antrum present). In this study, small preantral and antral horse follicles were removed with needles from ovarian tissue sliced at different thicknesses and classified by their location and other characteristics. The health of the harvested follicles was then examined in several ways (cell survival, toxic by-product levels, and certain types of gene control) to evaluate how slice thickness during follicle removal affects these structures. This study reports, for the first time, that tissue slice thickness influences the removal of late preantral and early antral follicles in horses and that the location of these follicles depends on their developmental stage and quality, with these follicles showing distinct levels of gene control.

## Introduction

The reproductive potential of most mammalian female species relies on a finite and non-renewable reserve of ovarian follicles, the functional units of the ovary that contain the oocyte and hormone-producing somatic cells (Grive 2020, Winship *et al.* 2020). Through the normal progression of follicular development, called folliculogenesis, primordial follicles are recruited from the ovarian reserve to grow and either ovulate an oocyte or undergo atresia and regress (McGee & Hsueh 2000, Tatone *et al.* 2008). Numerous factors influence the population of follicles within the ovarian reserve, including, but not limited to, hormones, metabolites, the initial ovarian reserve population, environmental conditions, medication use, and disease (for review, see Zhu *et al.* 2023). Furthermore, natural folliculogenesis and development of a competent oocyte are governed by both mechanical forces and molecular signals within the dynamic environment of the ovary (Biswas *et al.* 2022, Fiorentino *et al.* 2023). Understanding the complex mechanisms that regulate folliculogenesis and ovarian reserve is key to developing methods to preserve and extend the female reproductive lifespan. Assisted reproductive technologies (ARTs), such as ovarian tissue cryopreservation and *in vitro* culture, have emerged as promising strategies for preserving female fertility in humans (Takae & Suzuki 2019) and non-human animals (Campbell *et al.* 2014). Indeed, harvesting ovarian tissue fragments for cryopreservation and subsequent *in vitro* culture of isolated preantral and early antral follicles represents a safe and effective approach to fertility preservation in animals of high genetic value, endangered animal species, and oncological patients undergoing gonadotoxic medical treatments (Smitz *et al.* 2010, Michalczyk & Cymbaluk-Płoska 2021, Jensen *et al.* 2022). As such, developing techniques to reliably isolate morphologically normal, functionally intact, and viable follicles from ovarian tissue is imperative to improve these vital ARTs.

In this regard, the mare is an excellent model for exploring follicular isolation methods, given its unique dual-purpose, dual-benefit status. Not only can studies utilizing the mare provide insight into reproductive physiology in equine and other livestock species, but they also offer valuable information on women as well due to numerous shared reproductive similarities (for review, see Carnevale 2008, Mihm & Evans 2008, Ginther 2012, Bashir *et al.* 2016, Carnevale *et al.* 2020, Benammar *et al.* 2021). One such similarity is a heterogeneous distribution of follicles within the ovary (horse: Hyde *et al.* 2022*a*, Alves *et al.* 2018; human: Schmidt *et al.* 2003; cattle: González *et al.* 2023). This heterogeneous follicular distribution within the ovary suggests that these follicles migrate through different regions of the ovarian cortex, a phenomenon explained by ovarian plasticity (Woodruff & Shea 2011, Feng *et al.* 2017, Hyde *et al.* 2022*a*). In fact, a specific distribution pattern of preantral follicles has been identified in different regions of the mare ovary, such as the dorsal, medial, and ventral (near the ovarian fossa; Alves *et al.* 2015, 2016, Hyde *et al.* 2022*a*,*b*). These findings suggest that targeting specific areas of the ovary to collect follicles may help optimize follicular isolation techniques, thereby directly improving the efficiency of ARTs. Moreover, there is an urgent need for further studies to enhance the retrieval of follicles, especially in species with heterogeneous follicular distribution, such as horses (Alves *et al.* 2018, Hyde *et al.* 2022*a*).

Mechanical methods of follicle isolation, such as microdissection (removal of follicles from fragments of ovarian tissue using small needles and/or blades using stereomicroscopy), are appealing for the use of ARTs, as they allow for the collection of individual follicles without the use of potentially damaging enzymes (Nagashima *et al.* 2021, Babayev *et al.* 2022). Furthermore, microdissection can be used with ovarian tissue cryopreservation to collect follicles for *in vitro* culture (Lopes *et al.* 2020). However, there is little to no standardization of the optimal methodology for tissue fragmentation prior to microdissection, especially regarding the thickness of sliced tissue fragments. Creating thinner ovarian fragments (<0.5 mm) through slicing makes it easier to identify follicles for microdissection; however, there is a risk that early antral follicles, which often exceed 0.25 mm or 250 μm in diameter, may be damaged by the cutting blade or mechanical forces during fragmentation, potentially affecting isolation efficiency and quality assessments. Thicker ovarian fragment slices (≥0.5 mm) may mitigate the follicular damage and allow for recovery of higher numbers of intact antral follicles; however, follicles located in the middle of the ovarian tissue fragment may be difficult or even impossible to visualize for microdissection. As such, studies evaluating the effects of different thicknesses of ovarian tissue slices on the efficiency of follicle isolation and the quality of isolated follicles are crucial to evaluate this inherent trade-off.

In addition to the difficulty of collecting follicles from ovarian tissue, ovarian tissue processing (i.e., fragmentation and slicing) and follicle isolation can disrupt folliculogenesis, thereby affecting the efficiency of ARTs. While visual observation under a stereomicroscope with the aid of vital stains, such as trypan blue, can determine basic follicular morphology (i.e., developmental stage and normal or abnormal appearance) or the presence of intact cell membranes, the functionality of the follicle cannot be assessed without further analyses. Indeed, processes that manipulate ovarian tissue, such as slicing and isolation, regardless of ovarian tissue thickness, can disturb cellular metabolism and cell–cell communication mechanisms, trigger oxidative stress and epigenetic changes, and compromise membrane and DNA integrity (Demeestere *et al.* 2002, 2005, Gastal *et al.* 2017, 2019, Dey *et al.* 2024). As such, assessing the molecular effects of fragmentation and isolation on ovarian follicles is vital. Furthermore, evaluating epigenetic differences, such as the trimethylation of histone H3 lysine 4 (K4) and 9 (K9), across different developmental stages of isolated follicles can provide insight into the impact of fragmentation and isolation on equine ovarian follicles during the late secondary and early antral stages.

Given the impact of ovarian tissue processing on folliculogenesis, it is critical to investigate factors affecting the quality of isolated preantral and early antral follicles to optimize reproductive biotechnologies. Therefore, this study aimed to evaluate, for the first time in any species, the effects of microdissection on the quantity and quality of late preantral and early antral follicles obtained from different ovarian cortical regions (dorsal, medial, and ventral) and processed at two fragmentation thicknesses (0.25 vs 0.50 mm). To reach this goal, the following end points were evaluated: follicle number and diameter, follicular viability (using trypan blue and propidium iodide with Hoechst 33342), mitochondrial membrane potential (JC-1), and reactive oxygen species (ROS) levels (H2DCFDA). In addition, histone trimethylation patterns (H3K4me3 and H3K9me3) were analyzed to evaluate the impact of fragmentation and isolation on late secondary and early antral follicles. Our findings demonstrate that viable late preantral and early antral equine follicles can be successfully isolated. Moreover, these methods yield material suitable for evaluating key molecular and metabolic characteristics. This information will enable the collection of higher-quality, viable, and metabolically active follicles for use in *in vitro* techniques, such as follicular culture or ARTs, to increase success rates.

## Materials and methods

### Chemicals

Unless otherwise indicated, the chemical reagents used in this study were obtained from Sigma Chemical Co. (USA).

### Ovary collection and initial processing

**Figure 1:**
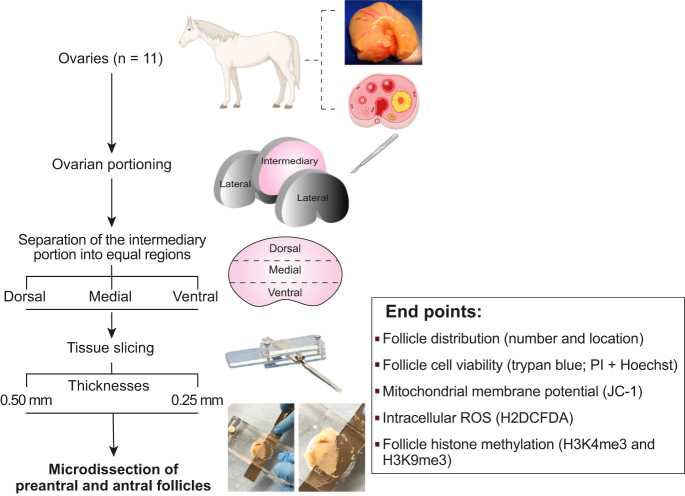
Experimental design for the microdissection of equine ovarian follicles and end points evaluated. One ovary was harvested from mixed-breed mares (*n* = 11) and sectioned into three ovarian portions (two lateral and one intermediary portion). The intermediary portion was further separated into three equally sized dorsal, medial, and ventral regions. The outermost cortical areas of these regions were sliced at 0.25 and 0.50 mm thicknesses using a Stadie-Riggs tissue slicer. All slices that contained visible preantral and antral follicles were subjected to microdissection to collect these follicles. The experiment was replicated 11 times, with each animal/ovary considered as one replicate. Microdissected follicles were evaluated for spatial distribution, viability, mitochondrial membrane potential, ROS, and histone trimethylation.

One randomly selected ovary from mixed-breed mares (*n* = 11), aged 2–12 years based on dental characteristics and of unknown reproductive status, was harvested from an equine slaughterhouse or post-ovariectomy in Rio Grande do Sul, Brazil, during the reproductive season. Any ovaries containing visible preovulatory follicles and/or corpora lutea were avoided. The ovaries were trimmed to remove excess adipose tissue and ovarian ligaments and then rinsed once in 70% alcohol (10 s), followed by a double wash in 0.9% saline solution (Chaves *et al.* 2008). The ovaries were then transported to the laboratory in 150 mL phosphate-buffered saline (PBS) at 4°C within 1 h (Gastal *et al.* 2017). Once in the laboratory, the ovaries were further trimmed to remove non-ovarian tissue and sectioned into three portions: two lateral and one intermediary portion (Alves *et al.* 2018; Fig. 1). The two lateral portions were discarded, and only the intermediary portion was considered for this study due to the high density of normal preantral follicles located in this portion (Hyde *et al.* 2022*a*). Next, the intermediary portion was separated into three equal regions: one dorsal region consisting of the greater mean curvature of the ovary, one medial region, and one ventral region containing the ovulation fossa. This experiment was replicated 11 times, with one intermediary portion per ovary being considered one replicate.

### Ovarian tissue slicing

Once the intermediary portions of all ovaries were sectioned into regions (i.e., dorsal, medial, and ventral), the outermost cortical piece of each ovarian region was sliced using a tissue slicer (Fig. 1). Each region generated four slices total: two 0.25 mm-thick slices were generated using an innovative, modified Stadie-Riggs tissue slicer (Federal University of Rio Grande; patent pending) and two 0.50 mm-thick slices were generated using a Stadie-Riggs tissue slicer (Thomas Scientific, USA). All slices were produced randomly. Afterward, each slice was examined using a stereomicroscope (Nikon SMZ645, Japan; ×100 magnification) for the presence of late secondary preantral and early antral/tertiary follicles. Only cortical slices that contained visible late secondary and/or early antral follicles were used for microdissection.

### Isolation and selection of preantral and antral follicles

**Figure 2:**
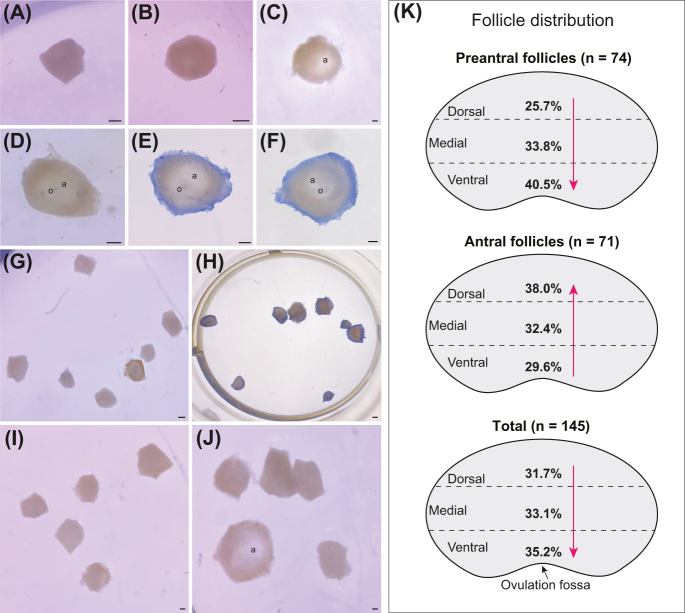
Microdissected preantral and antral equine ovarian follicles and spatial distribution within ovarian regions. (A, B, G, H, I) Preantral and (C, D, E, F) antral follicles and (J) a group of antral and preantral follicles after microdissection are displayed. (E, F, H) Representative images of follicles after trypan blue staining are shown. It is important to note that only the stromal cells around the follicles are stained positively (blue) for trypan blue, indicating damaged cell membranes and not live cells; meanwhile, the follicles remained unstained (intact cell membranes and live cells) and were used for downstream analyses. (K) The percentages of follicles distributed across each ovarian region (dorsal, medial, and ventral) within the intermediary portion are shown for each follicular developmental stage (preantral and antral) and combined (total). The arrows indicate spatial distribution trends within the ovary. The letter ‘a’ indicates antrum, and ‘o’ indicates oocyte. (A, B, C, D, E, F, G, H, I, J) Scale bar = 50 μm; magnification = ×20.

Late secondary preantral follicles and early antral follicles were visualized using a stereomicroscope (Nikon SMZ645; ×100 magnification) and manually microdissected using a 2.85 mm straight slit knife (52-2801; Surgical Specialties Corporation, USA; Fig. 2). All microdissected follicles were counted and visually classified based on developmental morphology (late secondary: visible, centrally located oocyte surrounded by two or more layers of cuboidal granulosa, intact basement membrane, no antrum, Fig. 2A, B, G, H, I; early antral: similar to late secondary but with an antrum, Fig. 2C, D, E, F). Follicular diameter was determined by measuring (using an eye piece ruler inserted into the stereomicroscope) and recording the greatest edge-to-edge widths on the horizontal and vertical axes of each follicle and then averaging these values (Aguiar *et al.* 2016). Only morphologically normal follicles (presence of intact basement membrane and an oocyte surrounded by granulosa cells that are well organized in two or more layers) were selected for the subsequent methodologies (Haag *et al.* 2013, Gastal *et al.* 2020).

### Pre-selection of follicles for membrane integrity

All isolated follicles were incubated in 0.4% trypan blue solution for 2 min at room temperature and visualized using stereomicroscopy (Nikon SMZ645; ×100 magnification). Membrane integrity of follicles (i.e., presence or absence of blue staining) was utilized as a marker of follicle health after isolation. All follicles were classified as viable (absence of blue staining in the follicle and/or oocyte, indicating intact follicular cell membranes) or non-viable (presence of blue staining in the follicle and/or oocyte, indicating damaged follicular cell membranes; Haag *et al.* 2013, Lopes *et al.* 2020). Examples of follicles exposed to trypan blue are shown (Fig. 2E, F, H), with only the stromal cells surrounding the follicle staining positive, while the follicle and oocyte remain unstained. Only live follicles were used for the following assessments.

### Characterization of follicles (viability, mitochondrial membrane potential, and reactive oxygen species)

Isolated follicles from every group were assessed using fluorescent probes for cell death (propidium iodide, PI), mitochondrial membrane potential (JC-1; Invitrogen, USA; Smiley *et al.* 1991), ROS (H2DCFDA; Fabbri *et al.* 2014), and the presence of DNA (Hoechst 33342; Invitrogen; Morais *et al.* 2023). When possible, the same follicle was subjected to two or more probes and was, therefore, included in multiple analyses. For follicle cell viability, follicles (*n* = 64) were incubated in PI 20 μM for 5 min at 37°C. For mitochondrial membrane potential, follicles (*n* = 63) were incubated in 5 μg/mL JC-1 for 30 min at 37°C (Gastal *et al.* 2019). To evaluate intracellular ROS, follicles (*n* = 60 for total ROS; *n* = 48 for ROS per viable cell) were incubated in 100 μM H2DCFDA for 30 min and then incubated in 20 μM PI for 5 min at 37°C. After the incubation period of each fluorescent probe, follicles were fixed in 70% alcohol at room temperature for 15 min and then transferred to PBS for up to 1 h at room temperature in a dark room for subsequent fluorescent image analysis. Next, follicles (*n* = 78) were mounted on slides, incubated in 16.2 μM of Hoechst 33342 (Hoechst) for 10 min, and analyzed under fluorescence microscopy (Olympus BX51l, Japan; ×400 magnification). Settings for evaluating fluorescence signals, such as laser energy, signal detection (gain), and pinhole size, were kept constant during all evaluations. A mercury lamp was utilized to excite all fluorophores. The emission characteristics for each fluorophore are detailed as follows: Hoechst emitted light in the range of 330–385 nm; JC-1 emitted in two distinct bands, 510–550 nm (indicating low mitochondrial membrane potential; green) and 580–620 nm (indicating high mitochondrial membrane potential; red); H2DCFDA produced emissions within the 510–550 nm range; and propidium iodide emitted light in the 580–620 nm range (Gastal *et al.* 2017). All pictures taken were analyzed using ImageJ software (ImageJ, version 1.53; National Institute of Health, USA) to determine fluorescence levels, and only the follicle was circled as the delimited area to exclude as much residual stromal cell tissue from the analyses as possible. It should be noted that, with the limitations of microdissection, it was not possible to completely eliminate peripheral stromal cells attached to theca cells without damaging the follicles. Therefore, delimiting the follicular area aimed to minimize fluorescent signals from more distant peripheral stromal tissue while still accounting for the putative paracrine signaling of the closely associated stromal cells. As such, all fluorescence signals obtained from the isolated follicles represent a combination of the oocyte, follicular cells, and stromal cells closely attached to the follicle. The following equations were used to determine fluorescence levels of each analyzed end point: percent cell viability = (1 − (PI/Hoechst)) × 100; fluorescence percentage of mitochondrial membrane potential = (JC-1 red fluorescence/(JC-1 green + red fluorescence)) × 100; intracellular ROS production for all cells = (H2DCFDA/Hoechst) × 100; and intracellular ROS production for viable cells = (H2DCFDA/(Hoechst − PI)) × 100.

### Quantification of histone trimethylation

Preantral and antral follicles (*n* = 18 per developmental stage) were fixed with 4% paraformaldehyde in PBS (pH 7.2) and subsequently dehydrated, diaphonized, and embedded in paraffin. Sections of the follicular samples (5 μm) were mounted on Superfrost Plus slides (Knittel Glass, Germany), deparaffinized with CitriSolv (Fisher Scientific, USA), and rehydrated in increasing concentrations of ethanol. Antigen retrieval was performed by incubating samples in 0.01 M sodium citrate buffer (pH 6) at 95–100°C for 5 min in a pressure cooker (Silva *et al.* 2023). After cooling to 37°C, follicle sections were washed in PBS and blocked for 1 h at room temperature using PBS containing 1% (w/v) bovine serum albumin. The slides were incubated overnight at 4°C with polyclonal primary antibodies for anti-H3K4me3 (1:1,000; ab8580; Abcam, UK) and anti-H3K9me3 (1:1,500; ab8898; Abcam). Negative control samples from mouse kidney were prepared by omitting the primary antibodies. Subsequently, slides were incubated with fluorescent donkey anti-rabbit IgG secondary antibodies (AlexaFluor® 488; 1:500; ab150073; Abcam) for 1 h at room temperature and then mounted on slides using two drops of fluorescent mounting medium with DAPI (ab104139; Abcam). Finally, the slides were visualized under a scanning microscope (Zeiss Apotome.2; Oberkochen, Baden-Württemberg, Germany; ×10 magnification) at excitation wavelengths of 405 and 488 nm. Images (1,024 × 1,024 pixels) were individually captured using a monochrome digital camera to measure fluorescence. Exposure gains and rates were kept constant across samples. Fluorescence levels were quantified in all preantral and antral follicles using ImageJ software (Guerreiro *et al.* 2018, Silva *et al.* 2023). To determine levels of follicular histone trimethylation, the following formulas were used: H3K4me3 fluorescence percentage = (H3K4me3 fluorescence (green)/DAPI fluorescence (blue)) × 100; H3K9me3 fluorescence percentage = (H3K9me3 fluorescence (green)/DAPI fluorescence (blue)) × 100.

### Statistical analysis

All statistical analyses were performed using SAS statistical software version 9.4 TS1M8 (SAS Institute, USA). The Shapiro–Wilk or Kolmogorov–Smirnov test was used to verify the normal distribution of the data set, and data for end points that were not normally distributed were transformed using rank or log_10_. Outliers were identified using Dixon’s test and excluded from the data set prior to statistical analyses. Non-frequency data were analyzed using one-way ANOVA, followed by Tukey’s *post hoc* test. Frequency data were analyzed using the chi-square test. Data were expressed as mean ± S.E.M. or percentages. A value of *P* < 0.05 (two-sided) indicated that a difference was significant, and *P* ≥ 0.05 and ≤0.1 indicated that a difference approached significance.

## Results

### Isolated follicles and membrane integrity

Out of 150 isolated follicles, only 5 follicles were morphologically abnormal and discarded, leaving a total of 145 normal equine ovarian follicles (*n* = 74 preantral; *n* = 71 antral) for downstream analyses. The mean diameter of preantral and antral follicles analyzed in this study was 173.6 ± 9.9 μm (range: 96–280 μm) and 331.8 ± 21.8 μm (range: 185–431 μm), respectively. All morphologically normal follicles were considered alive (intact follicular cell membranes) after staining with trypan blue (Fig. 2E, F, H).

### Follicular distribution

Percentages of follicular distribution within the intermediary portion and for each ovarian region were determined considering follicular developmental stage and total follicles (Fig. 2K). While statistical analysis could not be performed on this end point, differing trends were observed for the distribution percentages of follicles according to ovarian region. Preantral follicles showed increasing distribution from the dorsal to the ventral region, while antral follicles showed the opposite trend (increasing distribution from ventral to dorsal). When follicular developmental stages were combined, the total number of follicles showed an increasing percentage distribution from the dorsal to ventral regions.

**Figure 3:**
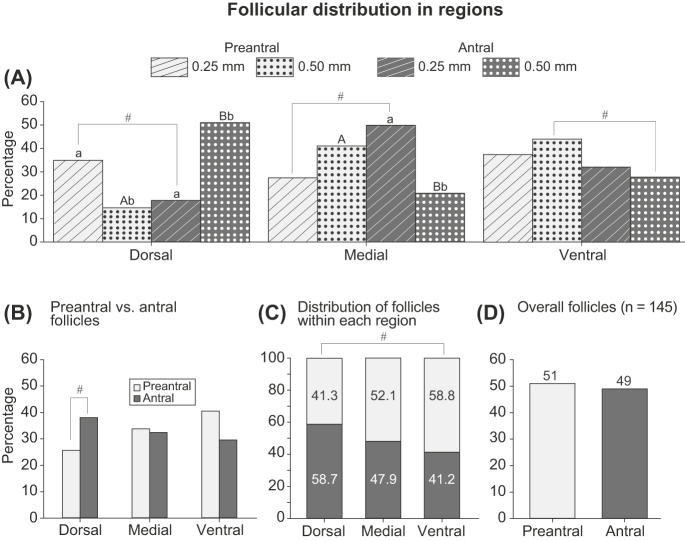
Percentages of preantral and antral follicles distributed in (A, B, C) different ovarian regions of the intermediary portion according to (A) slicing thickness and (B, C, D) with thicknesses combined. Numbers (C) within or (D) above each histogram box indicate the percentage of each follicular developmental stage. (D) Percentages of antral and preantral follicles, regardless of ovarian region and slicing thickness, are shown. ^a,b^Different lowercase superscripts indicate differences (*P* < 0.05 and *P* < 0.005 for preantral and antral follicles, respectively, in the dorsal region, and *P* < 0.01 for antral follicles in the medial region) between tissue slice thicknesses within the same follicular developmental stage and ovarian region. ^A,B^Different uppercase superscripts indicate differences (*P* < 0.0009 and *P* < 0.05 in the dorsal and medial regions, respectively) between preantral and antral follicles from the same ovarian region using the same slicing thickness. ^#^Indicates statistical tendencies between (A) preantral and antral follicles using the 0.25 mm slice thickness in the dorsal (*P* = 0.1) and medial (*P* = 0.06) regions and the 0.50 mm slice thickness in the ventral region (*P* = 0.1); (B) preantral and antral follicles in the dorsal region (*P* = 0.1), irrespective of ovarian tissue slice thickness; and (C) dorsal and ventral regions (*P* = 0.08) for both preantral and antral follicles, regardless of slice thickness.

When considering the dorsal region of the ovary, more (*P* < 0.05; Fig. 3A) preantral follicles were isolated from 0.25 mm slices than from 0.50 mm slices, while more (*P* < 0.005; Fig. 3A) antral follicles were isolated from the 0.50 mm slices. Moreover, when considering different developmental stages of follicles isolated from the same tissue slice thicknesses, more (*P* < 0.0009; Fig. 3A) antral follicles were isolated from the 0.50 mm slices, while there was a tendency for more (*P* = 0.1; Fig. 3A) preantral follicles to be isolated from the 0.25 mm slices. Within the medial region, more (*P* < 0.01; Fig. 3A) antral follicles were isolated from the 0.25 mm-thick tissue slices than from the 0.50 mm slices. Furthermore, more (*P* < 0.05; Fig. 3A) preantral follicles than antral follicles were isolated from the 0.50 mm-thick slices, while there was only a tendency for more (*P* = 0.06; Fig. 3A) antral follicles than preantral follicles to be isolated from the 0.25 mm slices. Finally, in the ventral region, there was a tendency for more (*P* = 0.1; Fig. 3A) preantral follicles than antral follicles to be isolated from 0.50 mm-thick slices. When ovarian tissue slice thicknesses were combined, there was a tendency (*P* = 0.1; Fig. 3B) for a greater percentage of antral follicles than preantral follicles to be isolated from the dorsal region. Furthermore, when analyzing all follicles isolated from each region (Fig. 3C), the dorsal region tended (*P* = 0.08) to have a lower percentage of preantral follicles when compared to the ventral region, with antral follicles showing the opposite trend. The developmental stages of all isolated follicles were evenly distributed when ovarian regions were combined (Fig. 3D).

### Viability of follicular cells

**Figure 4:**
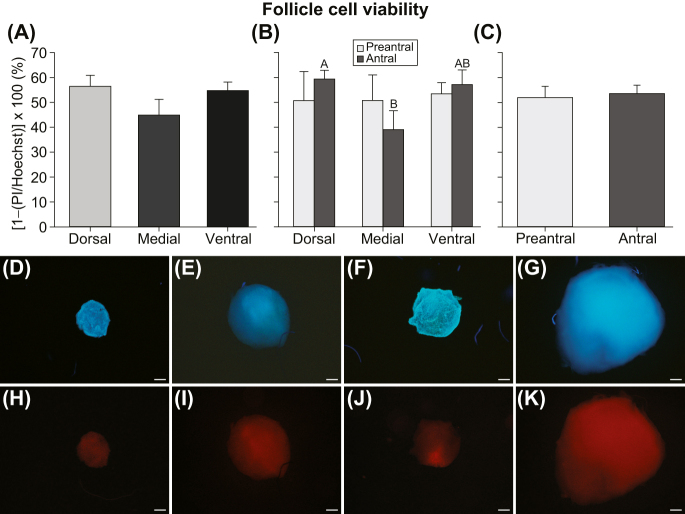
(A, B, C) Mean (±SEM) percentage of cell viability for microdissected follicles (*n* = 64) according to (A and B) different ovarian regions within the intermediary portion and (B and C) follicular developmental stages. Viability was considered regardless of (A) follicular developmental stage, (C) ovarian region, and (A, B, C) tissue slicing thickness. (D, E, F, G, H, I, J, K) Representative images of follicles stained with (D, E, F, G) Hoechst 33342 and (H, I, J, K) propidium iodide are shown. Both (D, E, H, I) preantral and (F, G, J, K) antral follicles are displayed. Percent cell viability = (1 − (propidium iodide/Hoechst 33342)) × 100. ^A,B^Different superscripts indicate differences within the same follicular developmental stage between ovarian regions. (A, B, C) PI, propidium iodide; Hoechst = Hoechst 33342. (D, E, F, G, H, I, J, K) Scale bar = 50 μm; magnification = ×40.

When follicular cell viability was classified for ovarian region and developmental stage (Fig. 4B), a greater (*P* < 0.03) percentage of antral follicles was found in the dorsal region compared to the medial region. However, no differences (*P* ≥ 0.05) were observed when evaluating the percentages of antral and preantral follicles within the same ovarian region (Fig. 4B), combining developmental stages of follicles (Fig. 4A), and comparing developmental stages, regardless of ovarian region (Fig. 4C). Furthermore, no difference (*P* ≥ 0.05) was found regarding the viability of follicular cells when comparing the two ovarian tissue slicing thicknesses (0.25 vs 0.50 mm) evaluated in this study (mean – 0.25 mm: 49.9 ± 5.9%; 0.50 mm: 54.3 ± 2.9%; range: 6.6–84.9%). Images of preantral and antral follicles stained with Hoechst (Fig. 4D, E, F, G) and PI (Fig. 4H, I, J, K) are shown.

### Mitochondrial membrane potential

**Figure 5:**
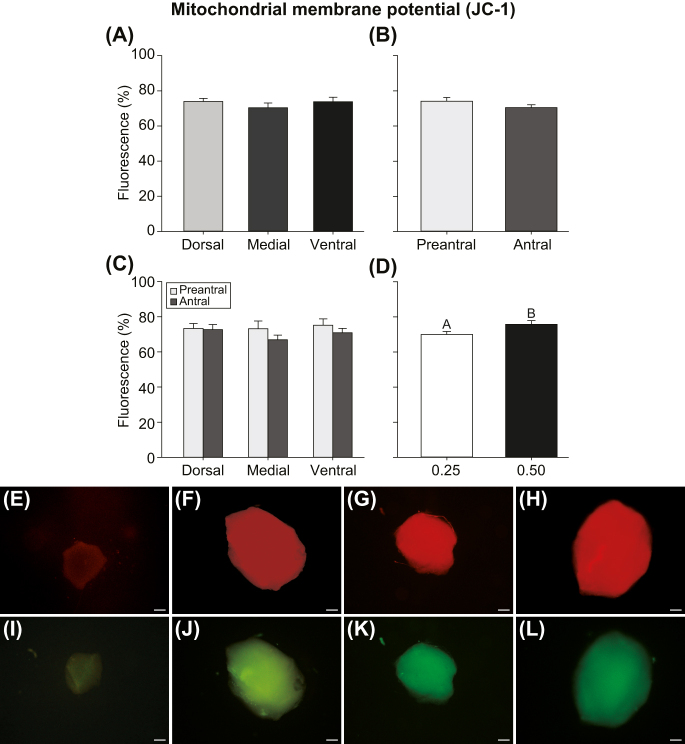
(A, B, C, D) Mean (±SEM) fluorescence percentage for mitochondrial membrane potential in microdissected follicles (*n* = 63), according to (A and C) different ovarian regions within the intermediary portion, (B and C) follicular developmental stage, and (D) tissue slicing thickness. Mitochondrial membrane potential was considered regardless of (A, B, C) tissue slicing thickness, (A and D) follicular developmental stage, and (B and D) ovarian region. (E, F, G, H, I, J, K, L) Representative images of follicles stained with JC-1 are shown. (E, F, G, H) Red staining indicates high mitochondrial membrane potential, while (I, J, K, L) green staining indicates low mitochondrial membrane potential. Both (E, F, I, J) preantral and (G, H, K, L) antral follicles are shown. Fluorescence percentage of mitochondrial membrane potential = (JC-1 red fluorescence/(JC-1 green + red fluorescence)) × 100. ^A,B^Different superscripts indicate differences between tissue slicing thicknesses regardless of ovarian region and follicular developmental stage. (E, F, G, H, I, J, K, L) Scale bar = 50 μm; magnification = ×40.

Mitochondrial membrane potential was evaluated using JC-1 (Fig. 5). A greater (*P* < 0.04) mitochondrial membrane potential was observed in follicles (preantral and antral combined) isolated from 0.50 mm-thick ovarian tissue slices when compared to follicles from 0.25 mm-thick slices, regardless of ovarian region (Fig. 5D). No differences (*P* ≥ 0.05) were observed in mitochondrial membrane potential when comparing different ovarian regions regardless of follicular developmental stage (Fig. 5A) and when follicular developmental stages were compared regardless of ovarian region (Fig. 5B). In addition, mitochondrial membrane potential was similar (*P* ≥ 0.05) between preantral and antral follicles within the same ovarian region and when considering the same follicular developmental stage between regions (Fig. 5C). Images of preantral and antral follicles with high mitochondrial membrane potential (red; Fig. 5E, F, G, H) and low mitochondrial membrane potential (green; Fig. 5I, J, K, L) are shown.

### Intracellular ROS production

**Figure 6:**
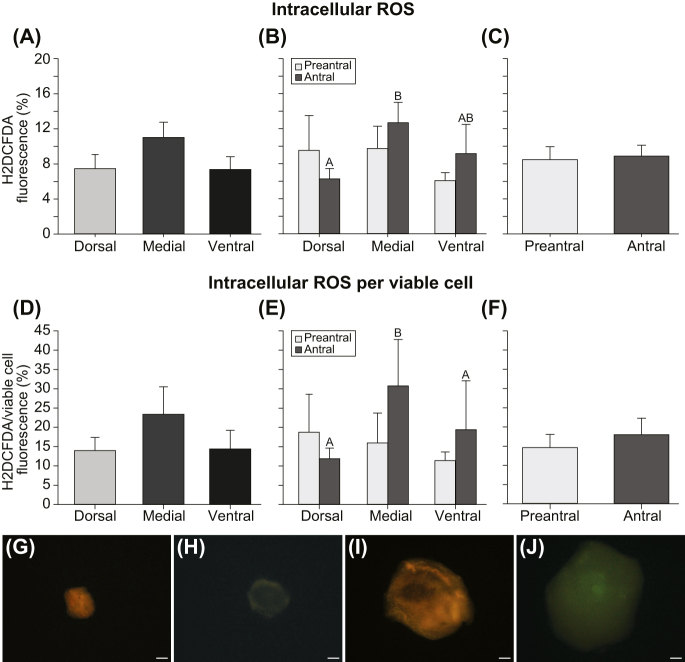
(A, B, C, D, E, F) Mean (±SEM) fluorescence percentage for intracellular ROS production for (A, B, C) all cells (*n* = 60 follicles) and (D, E, F) viable cells (*n* = 48 follicles) in microdissected follicles from the intermediary portion of the ovary. Intracellular ROS production was considered according to (A, B, D, E) different ovarian regions and (B, C, E, F) follicular developmental stage, regardless of tissue slicing thickness. Furthermore, ROS production was also considered regardless of (A and D) follicular developmental stage and (C and F) ovarian region. (G, H, I, J) Representative images of follicles stained with H2DCFDA are shown, with both (G and I) high and (H and J) low staining depicted. Both (G and H) preantral and (I and J) antral follicles are displayed. Intracellular ROS production for all cells = (H2DCFDA/Hoechst 33342) × 100; intracellular ROS production for viable cells = (H2DCFDA/(Hoechst 33342 − propidium iodide)) × 100. ^A,B^Different superscripts indicate differences between ovarian regions within the same follicular developmental stage. (G, H, I, J) Scale bar = 50 μm; magnification = ×40.

When intracellular ROS production was evaluated using H2DCFDA, follicular cells within antral follicles isolated from the medial ovarian region showed higher (*P* < 0.03) ROS production than those from the dorsal region (Fig. 6B). Furthermore, when intracellular ROS production was assessed in only the viable follicular cells, the cells from antral follicles isolated from the medial region had higher (*P* < 0.05) ROS production than those from both the dorsal and ventral regions (Fig. 6E). Meanwhile, the ROS production in all cells (Fig. 6B) and in only viable cells (Fig. 6E) did not differ (*P* ≥ 0.05) between preantral and antral follicles isolated from the same ovarian region. Similar (*P* ≥ 0.05) ROS production levels were observed when comparing all cells (Fig. 6A) and only viable cells (Fig. 6D) from follicles isolated from different ovarian regions, regardless of the follicular developmental stage. In addition, when comparing follicular developmental stages (preantral vs antral) regardless of ovarian region, no differences (*P* ≥ 0.05) were observed in ROS production levels for all follicular cells (Fig. 6C) and viable cells (Fig. 6F). Moreover, no differences (*P* ≥ 0.05) were observed in ROS production when comparing follicles isolated from 0.25 mm-thick slices to those isolated from 0.50 mm slices (mean – 0.25 mm: 9.8 ± 1.7%; 0.50 mm: 8.0 ± 1.2%; range: 1.7–34.9%). Follicles displaying high (Fig. 6G and I) and low (Fig. 6H and J) intracellular ROS production are shown.

### Histone trimethylation patterns

**Figure 7:**
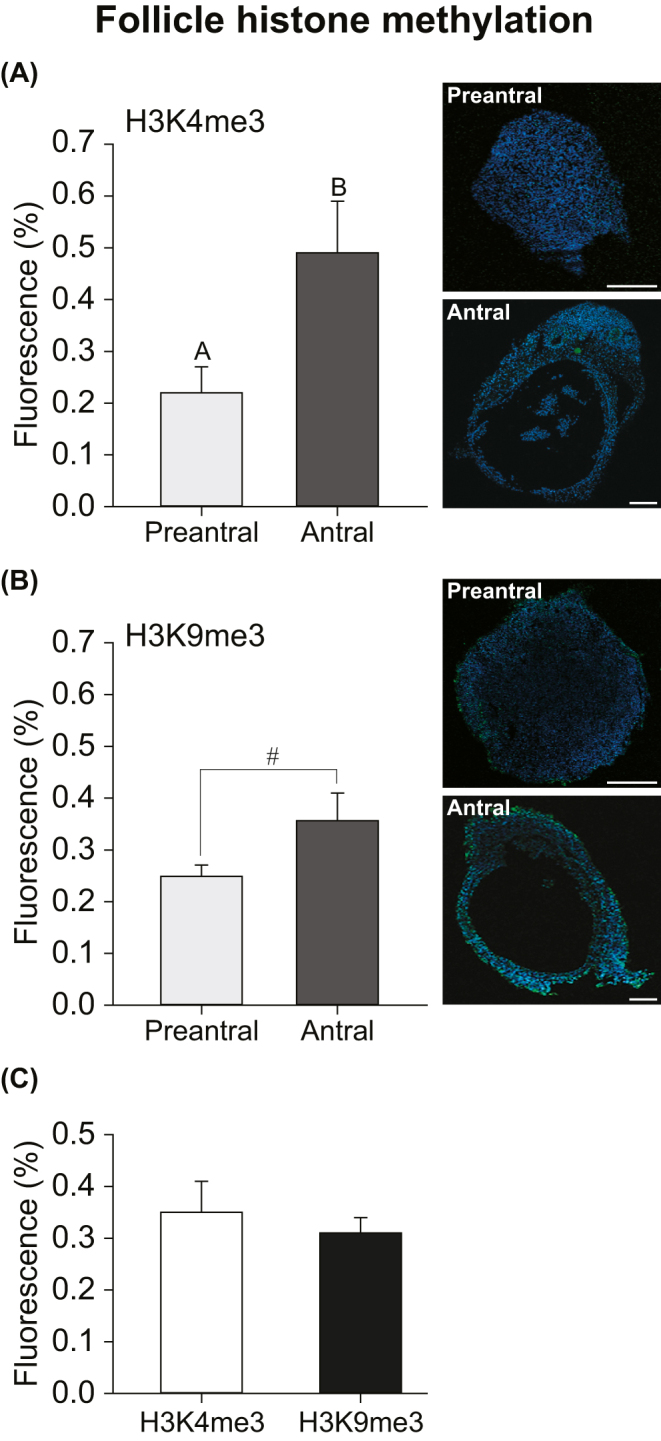
(A, B, C) Mean (±S.E.M.) fluorescence percentage of (A and C) H3K4me3 (*n* = 18 follicles) and (B and C) H3K9me3 (*n* = 18 follicles) according to (A and B) different follicular developmental stages and (C) with follicular stages combined in immunolabeled cells in microdissected follicles from the intermediary portion of the ovary. (A and B) Representative images of preantral and antral follicles immunolabeled for (A) H3K4me3 and (B) H3K9me3 are shown. H3K4me3 fluorescence percentage = (H3K4me3 fluorescence (green)/DAPI fluorescence (blue)) × 100; H3K9me3 fluorescence percentage = (H3K9me3 fluorescence (green)/DAPI fluorescence (blue)) × 100. ^A,B^Different superscripts indicate differences between follicular categories. ^#^Indicates a tendency (*P* = 0.09) between follicular categories. (A and B) Scale bar = 50 μm; magnification = ×10.

Antral follicles showed higher (*P* < 0.01) levels of H3K4me3 (Fig. 7A) and a tendency (*P* = 0.09) for higher levels of H3K9me3 (Fig. 7B) than preantral follicles, regardless of ovarian region or tissue slice thickness. Furthermore, when follicular developmental stages were combined, no differences (*P* ≥ 0.05) were observed between the trimethylation patterns (H3K4me3 vs H3K9me3; Fig. 7C). Representative images of preantral and antral follicles are shown for H3K4me3 (Fig. 7A) and H3K9me3 (Fig. 7B) under fluorescence microscopy.

## Discussion

This study reports, for the first time in the equine species, the isolation of late-stage preantral (secondary) and early antral (tertiary) follicles from ovarian tissue using mechanical isolation (microdissection). In addition, the present study details methodological enhancements that consider the spatial distribution of follicles within the dorsal, medial, and ventral regions of the intermediary portion of the ovary sliced at distinct thicknesses (0.25 and 0.50 mm). Finally, to characterize and assess the quality of the isolated follicles, parameters such as follicle number, morphology, diameter, viability, mitochondrial membrane potential, ROS levels, and histone trimethylation patterns (H3K4me3 and H3K9me3) were evaluated. A substantial number of follicles (*n* = 145) with intact membranes (a proxy for viability) were recovered, confirming the feasibility of this technique for equine ovarian tissue. The key findings from this study reveal that i) recovery of preantral and antral follicles within different ovarian regions is influenced by tissue slicing thickness; ii) the abundance of recoverable follicles (antral and preantral) varies among the ovarian regions; iii) preantral and antral follicle viability and ROS levels remain consistent between developmental stages, irrespective of tissue slicing thickness, although mitochondrial membrane potential is influenced by slicing thickness; iv) in antral follicles, viability and ROS levels vary depending on location within the ovarian regions; and v) epigenetic markers (H3K4me3 and H3K9me3) exhibit different patterns, depending upon follicular developmental stage. Altogether, these results indicate that successful preantral and antral follicle isolation depends on follicular spatial distribution trends observed throughout ovarian regions from the intermediary portion, according to different tissue slicing thicknesses. Furthermore, to the best of our knowledge, it is described for the first time in any species that antral follicle viability and oxidative stress levels are influenced by their location within the ovary.

The optimization of isolation techniques to recover viable, high-quality follicles from ovarian tissue is fundamental for advancing ARTs aimed at preserving female fertility and increasing reproductive lifespan. However, to date, only histological studies on early preantral follicles within ovarian tissue (Gonzalez *et al.* 2017, Alves *et al.* 2018, Hyde *et al.* 2022*a*) have provided detailed insights into the spatial distribution of follicles within the equine ovarian parenchyma. In this study, for the first time in any species, a substantial number of late preantral and early antral follicles were recovered from distinct regions (dorsal, medial, and ventral) of the intermediary portion, with tendencies for the greatest percentage of preantral follicles to be harvested from the ventral region and the greatest percentage of antral follicles to be harvested from the dorsal region. This observation aligns with previous findings from our group (Hyde *et al.* 2022*a*), which highlighted the interplay between ovarian location (regions and portions) and developmental stage on follicular spatial distribution in the mare. It has been shown that early preantral follicles (early secondary and younger) are more densely distributed in the intermediary ventral ovarian region (Hyde *et al.* 2022*a*), a finding mirrored by the late preantral follicles isolated in the current study. Furthermore, this observation shows that specific locations within the ovary (i.e., the intermediary dorsal region) can also be targeted to isolate high numbers of antral follicles.

In addition, tissue cut thickness influenced follicle recovery patterns within particular ovarian regions. While it was originally hypothesized that thinner tissue slices (0.25 mm) would enhance recovery of both late preantral and early antral follicles, more antral follicles were isolated from the 0.50 mm-thick slices in the dorsal region. Perhaps this region-specific variation occurs during tissue sectioning, where antral follicles (particularly those >0.25 mm) are damaged and therefore lost during the tissue-slicing process. Meanwhile, when isolated from the dorsal region, the antral follicles are more likely to be protected during thicker (0.50 mm) tissue slicing and, thus, be available for recovery via microdissection. In addition, in the dorsal region, thinner (0.25 mm) tissue slices enabled better visualization and, consequently, more effective recovery of the few preantral follicles found within this region. Interestingly, more early antral follicles were isolated from 0.25 mm-thick tissue slices from the medial region, perhaps indicating that these follicles are small enough (<0.25 mm) to avoid slicing damage. It has been suggested that preantral follicles migrate throughout the ovary in different patterns depending on the stage of development (primordial vs primary vs secondary) in several species (human: Woodruff & Shea 2011; mouse: Feng *et al.* 2017; horse: Alves *et al.* 2018, Hyde *et al.* 2022*a*). Furthermore, evidence from the mare shows that these follicles may also differentially migrate according to morphology (normal or abnormal; Alves *et al.* 2018, Hyde *et al.* 2022*a*,*b*). In the horse, evidence suggests that early preantral (primordial and primary) follicles migrate toward the ovarian geometric center (within the medial region of the intermediary portion), whereas early secondary follicles migrate away from the geometric center (Hyde *et al.* 2022*a*). Perhaps follicular migration continues as follicles develop into the antral stage, with early antral follicles in the medial region being less developed and therefore smaller than those in the dorsal region. These observations may reflect the dorsal region’s closer location to the richly vascularized ovarian medulla and pedicle than the medial region (Ginther 1992). This closer proximity to the more vascular areas of the ovary may better support these early antral follicles, which are known to be more sensitive to environmental fluctuations than their preantral follicle counterparts (Cadenas *et al.* 2017). However, future studies are needed to test this hypothesis and explore the interplay between the ovarian parenchymal environment and its influence on follicular development.

Characterization of isolated late preantral and early antral follicles revealed comparable follicle cell viability, mitochondrial functionality, and intracellular ROS levels between these two developmental stages. This shows that the mechanical isolation method performed in this study did not differentially affect the late preantral and early antral stages of follicular development, cementing microdissection as an appropriate method for isolating these follicles from the equine ovary. While the quality of preantral follicles was not affected by regional location within the ovary, antral follicles recovered from the dorsal region displayed superior follicle cell viability and lower intracellular ROS production than those isolated from the medial region. These findings may also be supported by the dorsal region’s closer proximity to the highly vascular ovarian medulla and pedicle (Ginther 1992). Alternatively, or perhaps concurrently, these patterns of antral follicles may reflect differential migration tendencies based on follicular quality. In a previous study by our group, morphologically abnormal early secondary follicles were found closer to the ovarian geometric center than their normal counterparts, suggesting that this migration ceases as follicles become less healthy or non-viable (Hyde *et al.* 2022*a*). Perhaps this is also mirrored in early antral follicles, with healthy (high viability, low ROS production) early antral follicles migrating toward the dorsal region of the intermediary portion (and maybe into the lateral portions; not evaluated in this study). Meanwhile, the less healthy antral follicles cease migration and remain within the medial region of the intermediary portion, closer to the ovarian geometric center. This may also explain the differential distribution of antral follicles across ovarian regions depending on the tissue slicing thickness.

Furthermore, when considering mitochondrial functionality, follicles isolated from thinner tissue slices (0.25 mm) showed reduced mitochondrial membrane potential compared to those from thicker tissue slices. While further studies need to be performed to evaluate this, perhaps the follicles residing within thicker ovarian tissue slices are more protected from the shearing forces of tissue slicing than those from thinner slices. Thus, the mitochondria within follicles may be more likely to be harmed and show lower mitochondrial membrane potential when ovarian tissue is thinly sliced versus thickly sliced. Considering all of this, for ART applications prioritizing viability, mitochondrial function, and oxidative stress parameters, slicing ovarian tissue at 0.50 mm and targeting the dorsal region within the intermediary portion appears optimal for healthy antral follicle recovery. In contrast, isolated preantral follicles maintained consistent metabolic characteristics across all regions, with higher percentages isolated from the medial and ventral regions, supporting their documented metabolic stability within the ovarian cortex (Cadenas *et al.* 2017, Sá *et al.* 2020, Silva *et al.* 2023). These findings highlight the need to develop tailored protocols for isolating particular follicle developmental stages.

This study also provides the first evidence of distinct epigenetic patterns between late preantral and early antral follicles in equine ovaries, suggesting key molecular events underlying this developmental transition. The trimethylation of K4 and K9 residues on histone 3 (H3K4me3 and H3K9me3) plays a pivotal role in gene regulation and cellular function (Krishnan *et al.* 2011, Black *et al.* 2012). Understanding these epigenetic differences is particularly valuable for optimizing ARTs that utilize late preantral and early antral follicles (Silva *et al.* 2021). In the current study, antral follicles displayed increased H3K4me3 (associated with transcriptional activation; Black *et al.* 2012, Park *et al.* 2020) and a tendency for higher H3K9me3 (associated with gene silencing and chromatin repression; Black *et al.* 2012, Ninova *et al.* 2019) levels compared to preantral follicles. These findings corroborate our previous reports that histone methylation patterns undergo dynamic changes during follicular growth *in vivo* (Silva *et al.* 2023). These results further suggest that the development of late preantral equine follicles into early antral follicles is tightly regulated by a balance between gene activation and repression. Epigenetic modifications, such as lysine trimethylation, alter electrostatic interactions between positively charged histone tails and DNA, modulating chromatin accessibility and ultimately affecting gene expression (Handy *et al.* 2011). Trimethylation of H3K4 is known to promote the expression of genes related to energy metabolism and cell survival (Mei *et al.* 2023), which may be important for the preantral-to-antral follicle transition. Meanwhile, the tendency for increased H3K9me3 may reflect the need to silence inappropriate gene expression and stabilize chromatin as the follicle continues to mature (Russo *et al.* 2013, Nicetto & Zaret 2019, Pang *et al.* 2023).

Previous studies have shown the importance of both trimethylation patterns in follicular development, including an increase in H3K4me3 in germinal vesicles (cats: Phillips *et al.* 2016) and H3K9me3 in oocytes (pigs: Bui *et al.* 2007) as follicles develop from the preantral to antral stages, corroborating the results shown in the current study (for review, see Pan *et al.* 2012). This trimethylation balance may reflect the physiological progression of follicular development, where chromatin stabilization in follicular cells (i.e., germinal vesicles, granulosa cells, and the oocyte) could become increasingly important as follicles advance to later stages (Nicetto & Zaret 2019, Li *et al.* 2022). Furthermore, other methylation patterns on these same H3 lysine residues, such as H3K4me2 and H3K9me2, have been reported to have dual regulatory potential and can mediate both gene activation and silencing (Pan *et al.* 2012, Liu *et al.* 2020). The coexistence of these opposing marks in antral follicles suggests a coordinated epigenetic program that balances gene activation and repression to support differentiation and safeguard genomic integrity (Becker *et al.* 2016, Silva *et al.* 2023). This finding further underscores the complexity of epigenetic regulation during folliculogenesis and warrants future studies.

In conclusion, this study reports, for the first time, the isolation of viable late-stage preantral (secondary) and early antral (tertiary) follicles in horses using mechanical isolation (microdissection), while also considering ovarian spatial distribution. Our optimized mechanical isolation technique successfully recovered viable, good-quality late preantral and early antral follicles from all three regions of the intermediary portion in equine ovaries. Regional quality variations were also observed, especially in antral follicles across different tissue slice thicknesses, suggesting a spatial influence on follicle quality and location at this developmental stage. Furthermore, early antral follicles exhibited distinct epigenetic patterns (H3K4me3 and H3K9me3) compared with late preantral follicles, highlighting, for the first time in horses, differential gene expression across this key phase of folliculogenesis. Altogether, these findings suggest a complex interplay between intrinsic developmental programming and extrinsic physical and spatial factors that serve to regulate folliculogenesis and affect the ability to recover late preantral and early antral follicles. Understanding how to best isolate a large number of healthy follicles is vital to improve the identification of follicular quality biomarkers, optimize follicular selection, and therefore increase the success rates of ARTs, such as *in vitro* culture and cryopreservation.

## Declaration of interest

The authors declare that there is no conflict of interest that could be perceived as prejudicing the impartiality of the research reported.

## Funding

This work was supported by the Coordination for the Improvement of Higher Education Personnel (CAPES, grant number 88887.617157/2021-00, and PDSE Program 88881.934282/2024-01). Ana Normélia Pereira de Morais received a doctorate scholarship from CAPES, Brazil.

## Author contribution statement

ANPM, GDAG, ASVJ, and ELG conceived the study. ANPM, AFBS, VABB, LG, ASVJ, and BRC curated the data. ANPM, LFL, LVSÑ, and ELG performed formal analysis. APRR, JRF, CDC, ASVJ, BRC, and ELG acquired funding and resources. ANPM, FLNA, GDAG, and ELG performed investigation. ANPM, LFL, LVSÑ, GDAG, MOG, ASVJ, and ELG designed the methodology. ANPM, ASVJ, and ELG administered the project. FLNA, GDAG, and ELG supervised the study. ANPM, KAH, FLNA, GDAG, and ELG wrote the original draft of the manuscript. ANPM, KAH, FLNA, GDAG, MOG, and ELG wrote, reviewed, and edited the manuscript.
